# Evaluating Vascular Hyperpermeability-inducing Agents in the Skin with the Miles Assay

**DOI:** 10.3791/57524

**Published:** 2018-06-19

**Authors:** James T. Brash, Christiana Ruhrberg, Alessandro Fantin

**Affiliations:** ^1^UCL Institute of Ophthalmology, University College London

**Keywords:** Biology, Issue 136, Vascular permeability, Miles assay, VEGF, edema, vascular leakage, Evans blue

## Abstract

The primary function of the vascular endothelium in vertebrate organisms is to serve as a barrier between the blood and each tissue of the body, whereby the permeability of the endothelium to blood cells, plasma macromolecules, and water can be adapted according to the physiological need. In certain diseases, cytokines and growth factors are released that target the endothelial barrier to transiently increase vascular permeability; however, their prolonged presence may cause chronic vascular hyperpermeability and thereby tissue-damaging edema. The Miles assay is an *in vivo* technique that allows researchers to study vascular hyperpermeability through the proxy measurement of vascular leakage. Here, we provide a detailed protocol on how to perform this procedure in the mouse, which is the most widely used model organism to study mammalian physiology and pathology. The procedure involves the intravenous injection of Evans blue dye to label the circulating albumin followed by multiple intradermal injections of permeability-inducing agents and vehicle control solutions into opposing flanks of the mouse. Consequently, Evans blue dye gradually leaks into the dermis, where it accumulates and can be extracted for quantification as leakage induced by the permeability-inducing agent relative to the vehicle. The Miles assay can be performed in wild type or genetically modified mouse models and may be combined with drug administration to study molecular mechanisms that regulate vascular permeability and identify agents/targets capable of inducing or blocking hyperpermeability.

**Figure:**
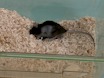


## Introduction

The primary function of the cardiovascular system is to enable the transfer of gases, nutrients, and waste products between the circulation and tissues in all organs. Blood vessels have organ-specific levels of basal permeability to permit such exchanges^1^. For instance, blood vessels in the kidney are highly permeable, whilst the blood brain barrier forms a tight, highly impenetrable interface^2^,^3^,^4^. The endothelial cells that form the inner lining of blood vessels provide a physical barrier between the circulation and underlying tissues and regulate vascular permeability in an organ-specific manner. However, certain stimuli cause a partial breakdown of the endothelial barrier to increase the fluid extravasation from the circulation into the interstitium above the basal levels^1^. Such hyperpermeability is observed, for example, at sites of tissue trauma, in inflammation, in tumors, during sepsis, in eyes with neovascular disease or in the brain and heart, when tissue ischemia occurs due to a stroke or myocardial infarction, respectively^5^,^6^,^7^,^8^. When chronically elevated, hyperpermeability leads to edema, which in turn causes tissue damage, such as loss of vision in eye disease^9^. Thus, modeling the vascular hyperpermeability response is desirable for understanding the mechanisms that increase endothelial permeability and to test the efficacy of agents designed to inhibit this.

The Miles assay is a well-established, commonly used and relatively simple technique that measures vascular leakage *in vivo* as a surrogate measurement of vascular hyperpermeability. Even though it does not take into account compounding factors that may increase vascular leakage independently of endothelial barrier regulation, such as blood pressure or blood flow, the Miles assay is generally thought to provide a reliable method to evaluate the permeability-modulating activity of substances and identify the signaling mediators that promote their activity. Accordingly, the Miles assay has been integral to numerous studies that have identified mediators of vascular hyperpermeability and their mechanisms of action^10^,^11^,^12^,^13^,^14^,^15^, such as the vascular endothelial growth factor VEGF-A that was originally identified as the vascular permeability factor VPF^16^.

Originally developed by Miles and Miles to study vascular permeability in guinea pigs^17^, their assay was subsequently adapted to using mice, which are now the model organism of choice to elucidate molecular mechanisms of vascular permeability regulation due to their exquisite suitability to genetic manipulation. Briefly, Evans blue dye is intravenously injected into adult mice and allowed to circulate for 30 minutes (Figure 1). Permeability-inducing agents versus vehicle control are then intradermally injected in multiple sites on opposing flanks of the mouse to induce vascular leak (Figure 1). Consequently, albumin-bound Evans blue dye extravasates and accumulates in the dermis (Figure 1). After humanely culling the mouse, Evans blue is extracted from the dermis and the level of vascular leak calculated as a ratio of test substance- to vehicle-induced optical density (Figure 2).

## Protocol

All animal work was carried out following UK Home Office and institutional Animal Welfare and Ethical Review Body (AWERB) guidelines.

### 1. Mouse Preparation

NOTE: Perform experiments on adult mice of at least 8 weeks of age, up to 6 months of age. In order to demonstrate technical reproducibility and consistent timing for each step of this procedure between different animals, use a minimum of 2 and maximum of 6 mice for each experimental session. To evaluate the effect of a mutation on a permeability-inducing substance in genetically modified mice, ideally, use 2 mutant mice and 2 littermate controls per experiments.

1. 24 h prior to stimulating vascular hyperpermeability, anesthetize mice using a suitable inhalation anesthetic such as isoflurane. Use 3% isoflurane for anesthesia induction until the righting reflex is lost and the mouse is unresponsive to external stimuli. Use 1.5% isoflurane for anesthesia maintenance (ensure that the mouse has constant respiratory rates). Under anesthesia, carefully shave both flanks of each mouse with an electrical shaver, avoiding injuries to the skin. NOTE: Anesthesia is used to avoid causing stress to the animal and to minimize movement during shaving, which may result in skin damage.
2. Return the shaved mouse to its home cage. For male mice, place each male into an individual cage after anesthesia recovery to prevent fighting and, therefore, damaging the skin. For female mice, return them in a group to their home cage. NOTE: If a fighting behavior is observed among male mice in the cage, before the procedure, separate them into individual cages for 3 days before the experiment to allow potential skin damage to heal.
3. Monitor the mice until they recover consciousness (usually within a few seconds) and move around the cage (usually within 1 min).

### 2. Intravenous Injection of Evans Blue Dye

1. In a laminar flow cabinet, prepare separate sterile solutions of the histamine inhibitor pyrilamine maleate (4 µg/µL in 0.9% saline) and of Evans blue dye (1% w/v in 0.9% saline). Sterilize by passing the solutions through a 22 µm filter.
2. Use a sterile 1 mL syringe with a 30 G needle to intraperitoneally inject each mouse with 10 µL pyrilamine maleate solution/gram of body weight (Figure 1A). To perform the injection, first, scruff the mouse and then tilt it so that the head is directed towards the ground and the abdomen is directed upwards. Inject in the lower quadrants of the abdomen away from the midline to avoid hitting the bladder. NOTE: The weight of mice between 8 weeks and 6 months of age usually ranges between 15 and 30 g. Pyrilamine maleate will inhibit the release of endogenous histamine, which would otherwise promote vascular leakage independently of the agent to be tested.
3. Place the mouse in a 37 °C heat chamber for 10 min to promote vasodilation. Alternatively, use a suitable heat lamp focused on the tail to promote vasodilation if the use of a heat lamp is permitted by the local ethical guidelines.
4. Move one mouse at a time to a mouse restrainer and rub the tail with 70% ethanol to clean the injection area and further promote vasodilation.
5. Use a sterile 1 mL syringe with a 30G needle to administer 100 µL Evans blue dye intravenously through the tail vein (Figure 1B). Immediately apply pressure to the injection site by holding the tail between a finger and the thumb to prevent bleeding. NOTE: Visualization of the tail vein can be improved by directing the lamplight to the injection area. Further detail on performing intravenous injections into the mouse tail vein can be found in a published protocol^18^.
6. Allow the dye to circulate for 30 min.

### 3. Stimulate Vascular Hyperpermeability

1. Using sterile solutions in a laminar flow cabinet, dilute the permeability-inducing agent of interest to a concentration that allows the final dose to be delivered in a volume of 20 µL. NOTE: In the example experiment shown in Figure 1-Figure 2, VEGFA is used to stimulate vascular hyperpermeability at a concentration of 2.5 ng/µL in PBS, yielding a total dose of 50 ng, and PBS is used as a vehicle control.
2. Load the permeability-inducing agent of interest and the vehicle control into 2 separate sterile 300 µL syringes with a 31 G needle. Load sufficient solution to inject each mouse with 20 µL of the solution in triplicate (i.e. prepare 60 µL of agent and vehicle per mouse, plus additional volume to account for the needle's dead volume).
3. Anesthetize the first mouse to be tested with isoflurane (3% for anesthesia induction until righting reflex is lost and the mouse is unresponsive to external stimuli, and 1.5% for anesthesia maintenance, making sure that the mouse has constant respiratory rates).
4. Intradermally inject 20 µL of the permeability-promoting agent into the flank of the mouse (Figure 1C). Ensure that the needle is at an angle of 15° to the skin. Check for the formation of a raised bubble within the skin which indicates a successful injection. Repeat the intradermal injection at 2 additional sites, a least 1 cm apart.
5. Turn the mouse to expose the 2nd flank and repeat triplicate injections with the control vehicle solution. Record on paper the position of each injection site to facilitate their identification for subsequent collection of skin samples. NOTE: Handle the mouse skin with caution at this stage; for example, avoid pinching the skin when performing intradermal injections.
6. Return the injected mouse to its home cage and monitor the mouse until it recovers consciousness (usually within a few seconds) and moves around the cage.
7. Whilst the first mouse recovers, immediately repeat steps 3.3 and 3.5 with the second mouse, and so on (up to 6 mice maximally per session). Keep a record of the time each mouse was injected to adjust the time at which to proceed to the subsequent step.

### 4. Quantification of Accumulated Evans Blue Within the Dermis

1. Cull each mouse by cervical dislocation 20 min after it has received the intradermal injections, observing the same order in which the mice were injected. NOTE: The duration of vascular leakage may vary according to the permeability-inducing agent used, and, therefore, the time between intradermal injection and culling may need optimizing.
2. Place each culled mouse on its back and pin its feet onto a cork board wrapped with a clean tissue (Figure 1D).
3. Using blunt scissors, make a vertical incision of approximately 3 - 4 cm from the lower abdomen up to the chest of the dead mouse. With forceps and a scalpel, tease away the skin from both flanks to reveal the inner side of the dermis and sites of Evans blue accumulation (Figure 1E). Pin down the loose skin and remove fat from regions around the leakage site with a scalpel. If required, take a representative photo to demonstrate the magnitude of Evans blue leakage into the skin.
4. Using forceps and a scalpel, excise skin regions comprising the leaked Evans blue dye for each site injected with the permeability-inducing agent or vehicle. NOTE: Take care to excise similarly sized regions of the skin to make sure that, at the subsequent Step 4.7, a similar portion of the formamide volume will be adsorbed by each skin sample, allowing the extracted dye to be diluted in a similar volume of remaining formamide. The injection map recorded in step 3.5 will help define the area to be excised.
5. Place each sample into a 1.5 mL tube and labeled it accordingly, making sure each sample rests on the bottom of the tube. Store the samples at - 20 °C until further use. If processing immediately, follow the steps below.
6. Dry the skin samples overnight by placing open tubes in an oven or into the well of a heating block at 55 °C.
7. To extract Evans blue dye, add 250 µL of deionized formamide to the samples in a fume hood, close the tubes. Make sure the skin samples are all covered by the formamide and incubate overnight in an oven or a heating block at 55 °C.
8. Centrifuge samples in a benchtop centrifuge at maximal speed (>10,000 x g) for 40 min.
9. In a fume hood, transfer 100 µL of the dye-containing supernatant from each sample into a separate well of a transparent, flat bottom 96-well plate (Figure 2A).
10. Measure peak Evans blue absorbance at 620 nm with a reference reading of 740 nm on a spectrophotometer. NOTE: 620 nm is the peak of Evans blue absorbance, whilst 740 nm is not absorbed by Evans blue and acts as a reference wavelength.
11. Average the absorbance readings of triplicate injections of the same agent or vehicle for each mouse to account for technical variability (Figure 2B).
12. Calculate the fold difference of readings from permeability-inducing agent versus vehicle control (Figure 2C).

## Representative Results

We used the Miles assay to assess the ability of VEGF-A to induce vascular leak relative to vehicle control (PBS) in wildtype C57/Bl6 mice. Here, we show a representative experiment using wildtype mice stimulated with intradermal injection of 20 µL solution containing 50 ng of VEGF-A in PBS or vehicle PBS only. A clear increase in Evans blue dye leakage in skin samples injected with VEGF-A compared to PBS was apparent *in situ* (Figure 1E) and after extraction of the dye in formamide (Figure 2A). Quantification of multiple experiments illustrates that 50 ng of VEGF-A significantly induces more Evans blue leakage than PBS alone (Figure 2B), with an average 3-fold increase in vascular leakage compared to vehicle (Figure 2C).

**Figure:**
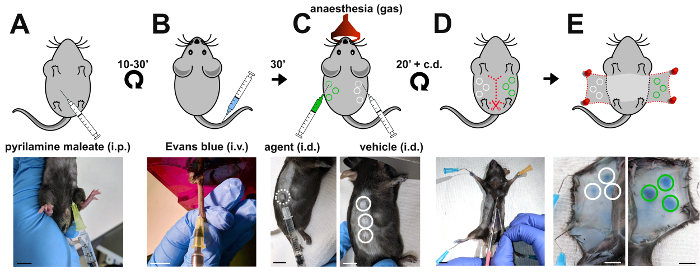

**Figure 1: Induction of vascular leakage in the Miles assay.** Schematic representation (top panels) and corresponding images (bottom panels) of the sequential steps when performing the Miles assay in the mouse. (**A**) Pyrilamine maleate is injected intraperitoneally to prevent the release of endogenous histamine 10 to 30 minutes prior to (**B**) intravenous injection of 100 µl 1% Evans blue into the tail vein of the mouse. (**C**) 30 min later, vascular leakage is induced by intradermal injection of the agent (50 ng of VEGF-A; green circles) versus vehicle control (PBS; white circles)**. **(**D, E**) To access sites of Evans blue accumulation, the mouse is culled 20 minutes after the intradermal injections and the skin of both flanks dissected and pinned down. i.p.: intraperitoneal; i.v.: intravenous; i.d.: intradermal; scale bars, 1 cm. Please click here to view a larger version of this figure.

**Figure:**
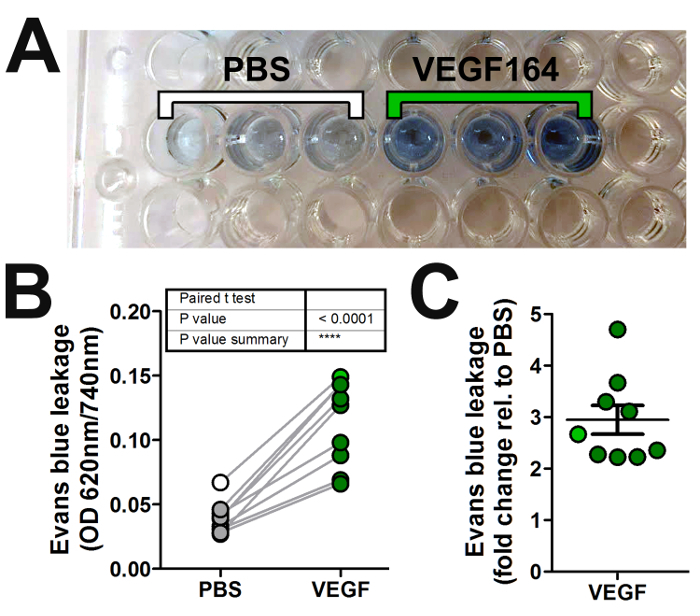

**Figure 2: Quantification of vascular leakage in the Miles assay.** (**A**) Evans blue dye is extracted in formamide from skin samples obtained from each intradermal injection in Figure 1 and loaded into a 96-well plate for absorbance reading with a spectrophotometer. (**B, C**) Graphical representation of the absorbance readings from multiple experiments by plotting either (**B**) the normalized absorbance values (optical density, OD) of both the permeability-inducing agent (VEGF-A; dark green circles) and vehicle (PBS; grey circles), or (**C**) the fold change in OD for the agent versus vehicle (error bars: SEM). The absorbance readings of the triplicate PBS and VEGF-A samples shown in (**A**) are averaged and shown in (**B, C**) as white or green circles, respectively. Please click here to view a larger version of this figure.

## Discussion

Since its first description in 1952^17^, the Miles assay has provided researchers with a relatively quick, simple, and reliable method for studying the molecular mechanisms of vascular hyperpermeability. For example, the Miles assay has been used to test the ability and/or potency of different agents to induce hyperpermeability^19^,^20^,^21^ or to test the efficacy of hyperpermeability blocking agents^8^,^22^,^23^. As another example, agents that induce vascular permeability have been tested in genetically modified mice and their littermate controls to determine the requirements of specific receptors and signal transducing proteins for ligand-induced hyperpermeability responses^10^,^11^,^12^,^13^,^14^,^15^,^20^,^23^. Several modified versions of the Miles assay have since been used, for example with respect to the tracer used. Thus, Evans Blue has been substituted in some studies with fluorescence-labeled dextrans of different sizes or microspheres^11^,^13^.

The main advantage of the Miles assay over other assays for investigating vascular hyperpermeability mechanisms is that it is comparatively easy to perform and does not require expensive equipment. Furthermore, as an *in vivo* technique, this assay models vascular leakage in the context of intact blood vessels, as opposed to measuring leakage through* in vitro* assays such as trans well flux assays or trans endothelial electrical resistance (TEER) assays, which focus, solely, on the endothelial monolayer. The alternative *in vivo* assays of vascular hyperpermeability may differ from the Miles assay described here by utilizing a different delivery route for the hyperpermeability-inducing agent or by analyzing vascular leakage at different sites, for example by systemic delivery of an agent via the tail vein followed by vascular leakage examination in the lungs or the trachea^11^,^13^,^15^. One limitation of the Miles assay is that the intradermal injection of certain agents may, in principle, also influence blood pressure and flow in addition to endothelial barrier disruption and, therefore, influence vascular permeability also indirectly. Nevertheless, this assay has recently been shown to measure vascular permeability induced by VEGF-A independently of effects on systemic blood pressure^15^. Another limitation of the Miles assay is that vascular beds in different tissues may respond differently to permeability-promoting agents, and results obtained with a Miles assay in the skin may, therefore, not be representative, for example, of what occurs in the lung or brain.

Animal's age and weight may influence the leakage observed in the Miles assay. To minimize the effect of these variables, researchers should use littermates or similarly sized and aged mice as controls. When assessing mouse mutants for a particular gene of interest, mice should be generated through a heterozygous versus heterozygous breeding strategy and wildtype littermates used as controls. Moreover, it is advisable to use quickly reversible gas anesthetics, such as isoflurane, to avoid vasoconstriction, which has been described for some anesthetic drug administered via parenteral injections^24^,^25^. The injection of Evans blue dye into the mouse lateral tail vein is a critical step in this protocol and can greatly influence the quality of the experiment and data. Thus, researchers must be competent in performing tail vein injections and will likely need prior experience or practice before beginning the Miles assay experiment. Alternatively, researchers could use other intravenous routes to deliver Evans blue, such as retro-orbital injection if they are experienced with it. Intradermal injection of permeability-promoting solutions can cause agent-independent damage to the skin. Therefore, intradermal injections should not exceed 20 µl in volume, should always be carried out in the presence of the histamine inhibitor and normalized to vehicle control injections.

The Miles assay has been used by many researchers to evaluate components of the VEGF-A signaling pathway, for example, because VEGF-A-induced vascular permeability causes ascites in cancer patients^16^ and vision-impairing edema in several neovascular eye disease^7^. Thus, we and others have used the Miles assay to compare VEGF-A induced vascular leakage in mice that have been genetically modified to lack specific components of the VEGF-A signaling cascade^12^,^13^,^14^,^15^,^20^,^23^. Our recent study using this approach revealed that the main pathological VEGF-A isoform, VEGF165, signals through a complex of VEGFR2 and NRP1, in which the NRP1 cytoplasmic domain promotes the ABL kinase-mediated activation of SRC family kinases to evoke a hyperpermeability response^14^. VEGFA also promotes blood vessel growth and could, therefore, be used therapeutically to restore blood flow to ischemic tissues if its hyperpermeability activities could be specifically inhibited. Research elucidating the molecular mechanism that steers VEGF-A responses of vascular endothelial cells towards vascular hyperpermeability might, therefore, identify pathways that can be selectively manipulated to inhibit pathological VEGF-A induced edema in diseases, such as cancer or ischemic eye disease. The Miles assay will undoubtedly continue to be a helpful method to underpin these and many other types of functional studies to elucidate molecular regulators and effectors of vascular hyperpermeability.

## Disclosures

The authors have no conflicting interests to declare.
